# Tubridge flow-diverting stent for treatment of unruptured intracranial complex aneurysms

**DOI:** 10.3389/fneur.2025.1584983

**Published:** 2025-06-16

**Authors:** Bao Juan, Xian Zhang, Yi Cao

**Affiliations:** Department of Cerebrovascular Disease, The Second Affiliated Hospital of Kunming Medical University Kunming, Kunming, China

**Keywords:** intracranial aneurysm, Tubridge flow diverter, complex intracranial aneurysms, brain disease, flow diversion

## Abstract

**Objective:**

To investigate the efficacy and safety of the Tubridge flow diverter (TFD) in treating unruptured intracranial complex aneurysms.

**Methods:**

A retrospectively was performed on consecutive patients with unruptured intracranial complex aneurysms treated with TFD at our institution between October 2019 and December 2022. Clinical data, including demographic characteristics, angiographic findings, and follow-up outcomes, were collected to assess postoperative and follow-up aneurysm occlusion rates. Complications and clinical outcomes were evaluated, with favorable outcomes defined as a modified Rankin Scale (mRS) score of 0–2.

**Results:**

A total of 72 patients harboring 75 aneurysms were included, with 47 aneurysms treated with TFD alone and 28 undergoing combined TFD and coiling. Fifty-six aneurysms in 53 patients underwent at least one digital subtraction angiography (DSA) examination. The median follow-up duration at the final visit was 156.00 days, yielding a successful aneurysm occlusion rate of 78.57% (44/56) and a median time to occlusion of 139.00 days. The complete occlusion rate was significantly higher in non-saccular aneurysms (92.86%, 13/14) than in saccular aneurysms (64.29%, 27/42). Severe stenosis (>50%) occurred in 3 of 55 stents (5.45%). Among 67 patients (5 lost to follow-up), 4 ischemic and 8 hemorrhagic complications were recorded, with 97.01% of patients achieving an mRS score of 0–1.

**Conclusion:**

Small-to-medium-sized aneurysms (maximum diameter <10 mm) can be effectively managed with TFD alone. TFD demonstrates minimal impact on branch vessels, and patients with mild in-stent stenosis may be monitored without intervention. The TFD is safe and effective for treating various types of unruptured intracranial complex aneurysms.

## 1 Background

Ruptured intracranial aneurysms represent the primary etiology of spontaneous subarachnoid hemorrhage, constituting nearly 85% of all cases and associated with substantial morbidity and mortality (1). Endovascular treatment has emerged as the preferred management strategy for unruptured intracranial aneurysms (2). However, the management of complex cerebral aneurysms remains substantially more complex, posing significant challenges for treatment with non-stent coil embolization (NSC), stent-assisted coil embolization (SAC), or balloon-assisted coil embolization (BAC) alone (3). By contrast, flow diverters (FDs) have gained widespread use in treating complex intracranial aneurysms due to their ability to effectively induce aneurysm occlusion while promoting durable reconstruction of the parent artery (4). In this study, we herein present the clinical data of the Tubridge flow diverter (TFD) and evaluate its efficacy and safety in the treatment of unruptured intracranial complex aneurysms.

## 2 Subjects and methods

### 2.1 Subjects

A retrospective analysis was conducted on 72 consecutive patients with unruptured intracranial complex aneurysms who underwent treatment with the Tubridge flow diverter (TFD) at our institution between October 2019 and December 2022.

#### 2.1.1 Inclusion criteria

1. Unruptured intracranial aneurysm;
2. Intracranial complex aneurysm diagnosed via cerebral angiography. Currently, there is no universally accepted clinical definition of complex intracranial aneurysms, which are typically characterized by anatomical features (e.g., large/giant aneurysms, wide neck, intra-aneurysmal thrombus, or calcification) and/or patient-specific conditions (e.g., advanced age, multiple comorbidities) (5). In this study, all patients underwent preoperative digital subtraction angiography (DSA) to delineate aneurysm location, size, and morphology, with inclusion criteria met if any of the following were present: (1) large or giant aneurysm (6); (2) aneurysm body-to-neck ratio ≤1.5 or neck width ≥4 mm; (3) wide-necked small-to-medium aneurysms precluding safe microcatheter navigation for coiling; (4) fusiform, pseudoaneurysmal, dissecting, or tandem multiple aneurysms without distinct necks; (5) recurrent aneurysms following prior endovascular treatment;
3. Male patients or non-pregnant female patients aged 18–75 years;
4. Treated with at least 1 TFD in each patient.

#### 2.1.2 Occlusion criteria

1. Acute ruptured aneurysm or aneurysm associated with an arteriovenous malformation;
2. Patients with contraindications or hypersensitivity to contrast agents or antiplatelet drugs (APDs);
3. Patients with severe systemic disorders (e.g., cardiac, hepatic, or renal insufficiency) or a life expectancy of <1 year;
4. Patients with severe mental illness precluding communication or functional outcome assessment due to disabilities;
5. Patients scheduled for major surgery within 3 months of enrollment, which may interfere with outcome evaluation;
6. Patients refusing to provide informed consent.

### 2.2 Methods

#### 2.2.1 Data collection

General clinical data were collected, including patient demographics (gender, age), aneurysm characteristics (location, number, size, morphology, neck width), surgical approach, and stent-covered branch vessels.

#### 2.2.2 Procedure

Digital subtraction angiography (DSA) was used to measure aneurysm dimensions, proximal/distal parent artery diameters, and select the appropriate Tubridge flow diverter (TFD; Microport, Shanghai, China). Vascular access was established using a bi-axial system comprising an 8F guide catheter and a 5F/6F U-track distal access catheter (Microport, Shanghai, China). Under microguidewire guidance, the stent delivery catheter was advanced to the distal parent artery, and the TFD was deployed along the catheter. The stent was released gradually and uniformly to ensure complete coverage of the aneurysm neck and secure apposition to the vessel wall. For stent-assisted coiling, a separate femoral artery sheath was used to introduce the coil embolization system, with aneurysms loosely packed with coils (dense embolization was not required).

#### 2.2.3 Evaluation of aneurysm occlusion

Aneurysm occlusion was assessed using the O’Kelly-Marotta (OKM) grading scale (7), which incorporates two parameters: contrast filling degree and stasis duration. Aneurysm filling is graded as: A (complete, >95% filling), B (incomplete, 5–95% filling), C (neck remnant, <5% filling), or D (no filling, 0%). Stasis grade is defined by contrast clearance timing: 1 (no stasis, cleared during arterial phase), 2 (moderate stasis, cleared before venous phase), 3 (significant stasis, persistent into venous phase). Combined, these yield 10 grades (A1–A3, B1–B3, C1–C3, D), with A1 to D indicating increasing occlusion. OKM grade D denotes complete occlusion, while grades C and above (C1, C2, C3, D) signify successful occlusion.

#### 2.2.4 Perioperative medications

Patients received dual antiplatelet therapy (DAPT) for 3–5 days preoperatively: 300 mg aspirin + 75 mg clopidogrel (or 90 mg ticagrelor bid for clopidogrel intermediate/slow metabolizers). DAPT was continued for 4–6 weeks, followed by 100 mg aspirin + 75 mg clopidogrel (or 90 mg ticagrelor) for 3 months, then switched to lifelong monotherapy with 100 mg aspirin.

#### 2.2.5 Complications assessment

Perioperative and follow-up complications were recorded, including hemorrhagic complications: Aneurysm rupture, guidewire-induced intraparenchymal hematoma, etc. Ischemic complications: Intraoperative thrombosis, postoperative symptomatic stroke, in-stent stenosis. DAPT-related complications: Gastrointestinal bleeding, epistaxis, gingival bleeding, non-pathological vaginal bleeding, massive skin bruising.

#### 2.2.6 Follow-up

Follow-up data included intervals, adverse events, and angiographic outcomes. Final clinical follow-up occurred in April 2023, with outcomes evaluated using the modified Rankin Scale (mRS). An mRS score of 0–2 was defined as a favorable outcome.

#### 2.2.7 Angiographic assessment

Pre-and post-treatment DSA images were independently reviewed and validated by two neurointerventionalists.

### 2.3 Statistical analysis

Statistical analyses were performed using IBM SPSS 26.0, R 4.3.3, and Zstats 1.0.^1^ Normally distributed data were reported as mean±standard deviation, while non-normally distributed data used median (25th–75th percentile). Categorical variables were summarized as frequencies (*n*, %). Group comparisons employed independent samples *t*-tests (normal data), Mann–Whitney *U* tests (non-normal data), chi-squared tests, or Fisher’s exact tests (categorical data). Correlations were analyzed via rank correlation, binary logistic regression, or partial correlation. Multivariate logistic regression assessed baseline predictors of complete occlusion, while univariate Cox regression and Kaplan–Meier curves evaluated time-dependent risk effects. Statistical significance was set at *p* < 0.05.

## 3 Results

### 3.1 Baseline patient and aneurysm characteristics

A total of 72 patients were enrolled, with a mean age of 54.0 ± 9.4 years, including 28 males (38.9%) and 44 females (61.1%). A total of 75 aneurysms in these patients were treated with Tubridge flow diverter (TFD) implantation: 69 were single aneurysms, and 3 were tandem multiple aneurysms located on the same parent artery. Seventy-three TFDs were deployed, including one patient with bilateral internal carotid artery aneurysms who received one stent per side. The median maximal aneurysm diameter was 4.20 mm [interquartile range (IQR), 3.20–7.68 mm], and all aneurysms were classified as wide-necked, with a mean neck width of 5.29 ± 3.77 mm. Detailed aneurysm characteristics are presented in Table 1.

**Table 1:** The baseline characteristics of the patients.

Characteristic	Patients (*N* = 75)
Age, mean (SD), year	54.0 (9.4)
Female	44 (61.1%)
Aneurysm size (mm)	
<5 mm	43 (57.33%)
≥5 and <10 mm	21 (28.00%)
≥10 and <25 mm	8 (10.67%)
≥25 mm	2 (2.67%)
Aneurysm type	
Saccular aneurysm	56 (74.67%)
Dissecting aneurysms	7 (9.33%)
Fusiform aneurysm	8 (10.67%)
Blood blister-like aneurysm	4 (5.33%)
Aneurysm location	
Internal carotid artery-C1	7 (9.33%)
Internal carotid artery-C4	4 (5.33%)
Internal carotid artery-C5	5 (6.67%)
Internal carotid artery-C6	39 (52.00%)
Internal carotid artery-C7	12 (16.00%)
Middle cerebral artery	1 (1.33%)
Vertebral artery-V4	7 (9.33%)

### 3.2 Immediate aneurysm occlusion

The technical success rate was 98.63% (72/73), with only one case experiencing stent shortening and proximal migration into the aneurysm during deployment. The immediate postoperative successful occlusion rate for the 75 aneurysms was 17.33% (13/75). Of these, 47 aneurysms were treated with TFD alone, and 28 underwent combined TFD and coil embolization.

### 3.3 Angiographic follow-up

#### 3.3.1 Overall angiographic results

Fifty-three patients with 56 aneurysms underwent at least one digital subtraction angiography (DSA) evaluation. The median time to first follow-up was 109.00 days [interquartile range (IQR), 96.00–170.50 days], during which the complete occlusion rate was 64.29% (36/56) and the successful occlusion rate (OKM grade C or higher) was 71.43% (40/56). At the final follow-up (median, 156.00 days; IQR, 103.50–266.50 days), the complete and successful occlusion rates were 71.43% (40/56) and 78.57% (44/56), respectively. The median time to successful aneurysm occlusion was 139.00 days (IQR, 100.00–200.00 days).

#### 3.3.2 Results of aneurysms treated by different procedures

Of the 56 aneurysms with angiographic follow-up, 33 were treated with TFD alone and 23 with TFD plus coiling. At the final follow-up, the TFD-only group achieved a complete occlusion rate of 60.61% (20/33) and a successful occlusion rate of 63.63% (21/33). In contrast, the TFD-coiling group demonstrated a complete occlusion rate of 86.96% (20/23) and a 100% successful occlusion rate (23/23). Univariate analysis revealed a significant association between coil use and complete occlusion (*χ*^2^ = 8.59, *p* = 0.003), with the coiling group achieving a significantly higher complete occlusion rate (100%) compared to the non-coiling group (63.64%). No statistical differences were observed in other baseline variables, including age, sex, or aneurysm size classification (*p* > 0.05) (Table 2).

**Table 2:** Univariate analysis of patients with incomplete vs. complete aneurysm occlusion.

Variables	Total (*n* = 56)	0 (*n* = 12)	1 (*n* = 44)	Statistic	*P*-value
Age, Mean ± SD	53.13 ± 9.43	54.36 ± 5.82	52.81 ± 10.20	t = 0.48	0.631
Sex, *n* (%)				*χ*^2^ = 0.00	0.984
Male	17 (32.08)	3 (27.27)	14 (33.33)		
Female	36 (67.92)	8 (72.73)	28 (66.67)		
Aneurysm size (Group), *n* (%)				–	0.079
<5	31 (55.36)	10 (83.33)	21 (47.73)		
≥10	10 (17.86)	0 (0.00)	10 (22.73)		
5–10	15 (26.79)	2 (16.67)	13 (29.55)		
Aneurysm type group, *n* (%)				*χ*^2^ = 3.29	0.070
Saccular	14 (25.93)	0 (0.00)	14 (32.56)		
Non-saccular	40 (74.07)	11 (100.00)	29 (67.44)		
Treatment, *n* (%)				*χ*^2^ = 8.59	**0.003**
TFD	33 (58.93)	12 (100.00)	21 (47.73)		
TFD + coiling	23 (41.07)	0 (0.00)	23 (52.27)		

t, *t*-test; *χ*^2^, Chi-square test; −, Fisher exact. SD, standard deviation. *p* < 0.05 indicated a statistically significant difference in the effect of coiling on occlusion rates.

In all subgroups—overall cohort, TFD-alone, and TFD-coiling—the final follow-up complete occlusion rate was significantly higher than the immediate postoperative rate (*p* < 0.05), underscoring the time-dependent efficacy of TFD in promoting aneurysm occlusion.

The crude model of Multivariate Logistic Regression demonstrated a significant association between coil use and complete occlusion (OR = 4.33, 95% CI = 1.07–17.57, *p* = 0.040). After adjusting for age, the effect trended toward significance (adjusted OR = 0.28, 95% CI = 0.07–1.15, *p* = 0.078). Subgroup analyses for aneurysm size and type were excluded from the final models due to imbalanced data distribution across subgroups. When adjusting for aneurysm type, the independent association of coil use with complete occlusion remained significant (adjusted OR = 5.23, 95% CI = 1.19–22.88, *p* = 0.028) (Table 3).

**Table 3:** Multivariate logistic regression analysis of factors influencing complete occlusion rate.

Variables	Crude		Age		Aneurysm type	
	OR (95% CI)	*P*-value	OR (95% CI)	*P*-value	OR (95% CI)	*P*-value
Treatment						
TFD	1.00 (Reference)		1.00 (Reference)		1.00 (Reference)	
Coiling	4.33 (1.07–17.57)	**0.040**	3.61 (0.87–15.02)	0.078	5.23 (1.19–22.88)	**0.028**

OR, Odds Ratio; CI, Confidence Interval; Aneurysm type, Saccular or non-saccular. *p* < 0.05 indicated a statistically significant difference in the effect of coiling on occlusion rates.

**Figure 1:**
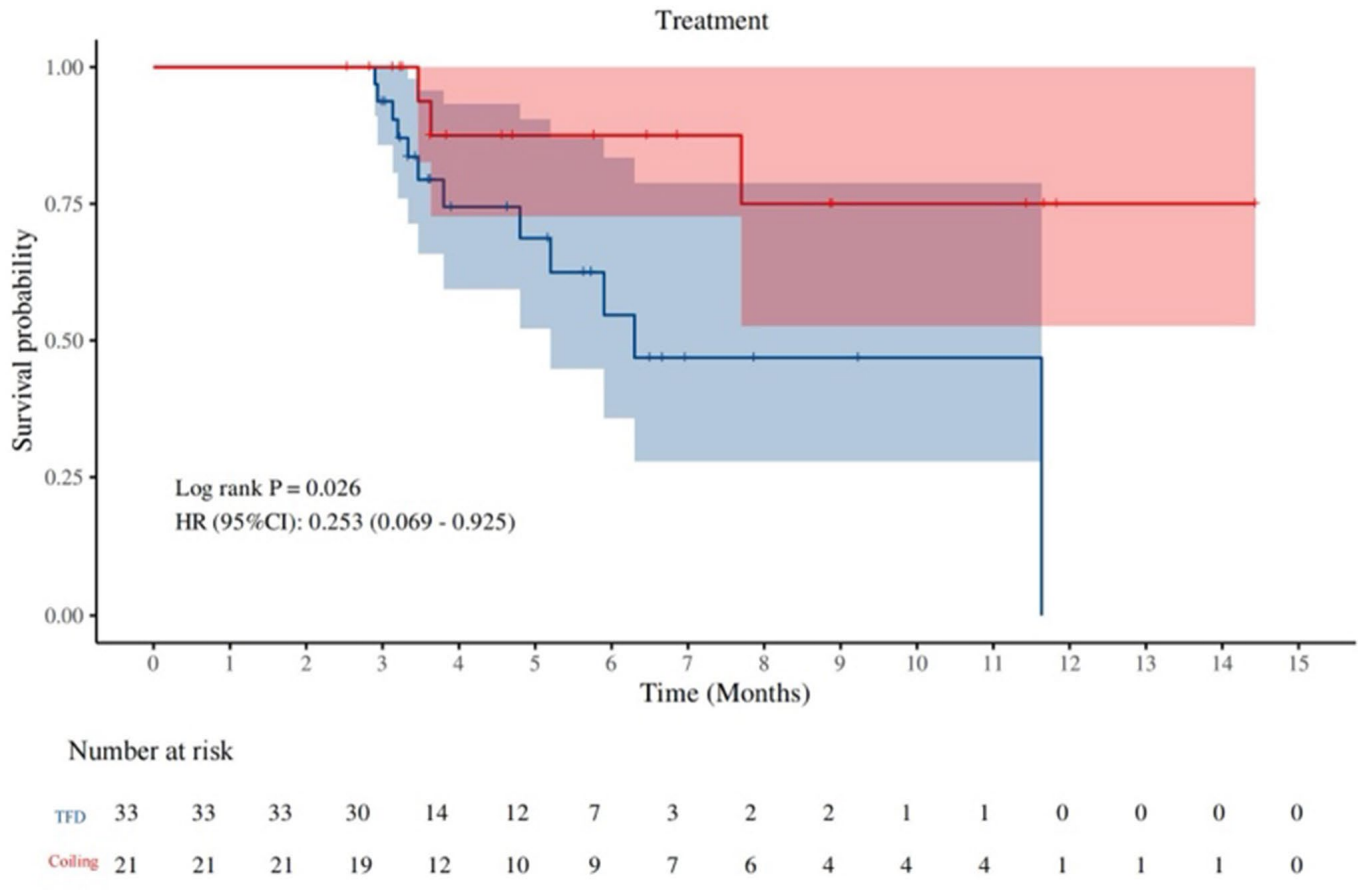
Time-dependent differences in complete occlusion rate (survival probability) between patients treated with and without coils.

The Kaplan–Meier curve demonstrated a significant time-dependent difference in complete occlusion rates (survival probabilities) between the coil-using and non-coil groups, with the survival probability curve for the coil-using group consistently positioned above that of the non-coil group, indicating a higher complete occlusion rate over time. Log-rank test results confirmed statistical significance in the between-group difference (*p* = 0.026), with a hazard ratio (HR) of 0.253 [95% confidence interval (CI): 0.069–0.925], reflecting a reduced risk of incomplete occlusion in the coil-using group (Figure 1).

#### 3.3.3 Results of aneurysms with different maximal diameters

Among the 56 aneurysms with angiographic follow-up, those with a maximum diameter <5 mm had a complete occlusion rate of 64.52% (20/31) and a successful occlusion rate of 67.74% (21/31). Aneurysms measuring 5–10 mm in diameter achieved both complete and successful occlusion rates of 86.67% (13/15). All 10 aneurysms ≥10 mm (treated with TFD-coiling) demonstrated a successful occlusion rate of 100% (10/10), with a complete occlusion rate of 70.00% (7/10). No significant intergroup difference in occlusion rates was observed at final follow-up between treatment groups for small-to-medium aneurysms (<10 mm; Table 4).

**Table 4:** Value of aneurysms with different maximal diameters.

Diameter	OKM grade	A1	A3	B1	B3	C1	C2	C3	D	*p*-value
<5 mm	TFD	2	5	1	2	0	0	1	16	0.274
	TFD with coiling	0	0	0	0	0	0	0	4	
5 mm–10 mm	TFD	1	0	0	1	0	0	0	4	0.143
	TFD with coiling	0	0	0	0	0	0		9	
≥10 mm	TFD with coiling	0	0	0	0	1	1	1	7	–

#### 3.3.4 Results of different types of aneurysms

Of the 56 aneurysms, 42 were saccular, with a median follow-up of 126 days, complete occlusion rate of 64.29% (27/42), and successful occlusion rate of 71.43% (30/42). Five dissecting aneurysms (median follow-up: 139 days) achieved 100% complete occlusion (5/5). Among six fusiform aneurysms (median follow-up: 106 days), the complete and successful occlusion rates were 83.33% (5/6) and 100% (6/6), respectively. All three blood blister-like aneurysms (median follow-up: 172 days) were completely occluded (3/3). At final follow-up, non-saccular aneurysms exhibited a higher complete occlusion rate (92.86%, 13/14) compared to saccular counterparts.

#### 3.3.5 In-stent stenosis

Fifty-five stents were implanted in 53 patients, with 18 (32.73%) demonstrating varying degrees of in-stent stenosis [median detection time: 109.00 days (103.00–189.00 days)]. Mild stenosis (<25%) occurred in 13 stents (23.64%). One patient with severe parent artery stenosis (due to proximal stent migration into the aneurysm) remained asymptomatic due to collateral compensation via the anterior communicating artery and anterior cerebral artery branches. Moderate stenosis (25–50%) was observed in two stents (3.64%), with a moderate-to-severe (>50%) stenosis rate of 5.45% (3/55); moderate stenosis improved in subsequent follow-ups. No patients developed symptomatic stenosis.

## 4 Complications

### 4.1 Perioperative complications

Perioperative complications occurred in 6 of 72 patients (8.33%, Table 5). One patient developed cerebral infarction due to in-stent thrombosis 3 days postprocedure, attributed to inadequate stent apposition based on angiographic analysis.

**Table 5:** Perioperative complications.

Complications	*n* (%)	Time
The stent shortened and fell	1 (1.39%)	Intraoperative
Dissection	3 (4.17%)	Intraoperative
Retroperitoneal hematoma	1 (1.39%)	Postoperative
In-stent occlusion and cerebral infarction	1 (1.39%)	3 days after surgery

### 4.2 Complications during follow-up

One patient with a middle cerebral artery aneurysm treated with TFD developed in-stent occlusion and cerebral infarction 1 month postdischarge after self-discontinuing dual antiplatelet therapy (DAPT). During follow-up, two patients experienced 1–2 transient ischemic attack (TIA) episodes without persistent neurological deficits.

No procedure-related aneurysm rupture occurred. Three patients experienced non-procedural hemorrhagic complications: one patient with a vertebral artery V4 segment aneurysm developed right limb motor dysfunction and left parietal-occipital lobe hemorrhage on CT 10 days postdischarge; two patients presented with mild headaches following minor localized subarachnoid hemorrhage in the frontal lobe ipsilateral to the treated artery. Additionally, five extra-procedural bleeding events were observed: three cases of gingival bleeding and two of epistaxis, all considered related to DAPT.

## 5 Clinical outcomes

**Figure 2:**
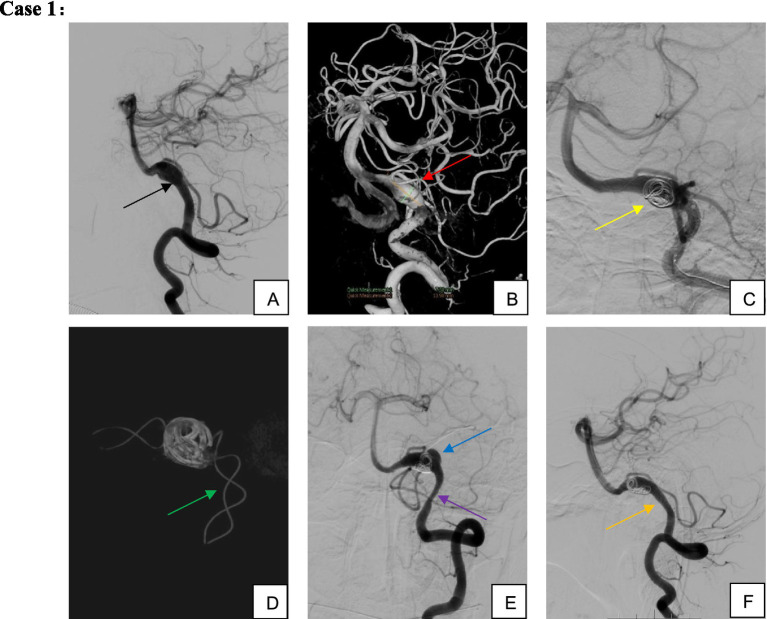
Male, 51-year-old, dissecting aneurysm of segment V4 of the left vertebral artery. **(A,B)** Left vertebral artery segment V4 dissecting aneurysm involving the left posterior inferior cerebellar artery; **(C,D)** TFD implantation associated with partial coil embolization; **(E)** Aneurysm recurrence and proximal stenosis noted 3 months postoperatively; **(F)** Without additional intervention, the patient was maintained on single-aspirin therapy for observation. By 9 months postoperatively, the aneurysm demonstrated complete healing, proximal stent stenosis spontaneously resolved, and the posterior inferior cerebellar artery remained patent.

**Figure 3:**
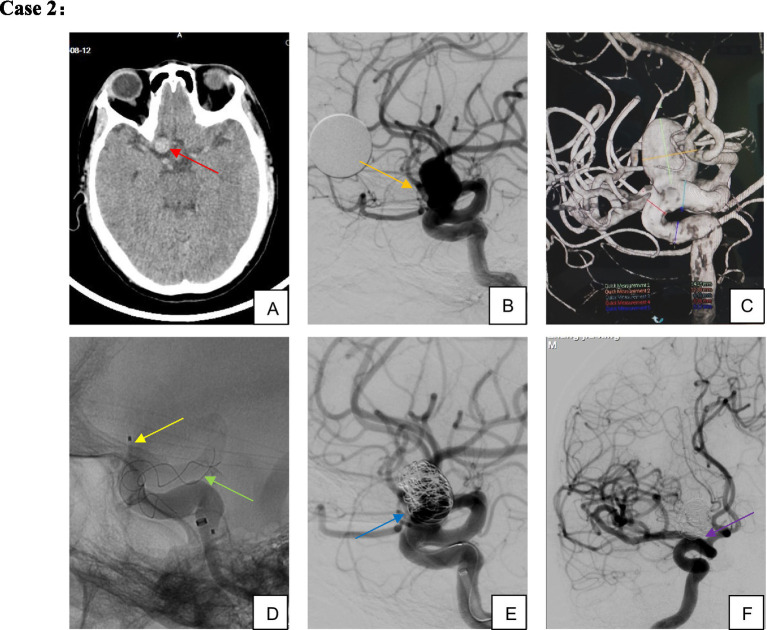
Male, 54-year-old, large aneurysm of the ophthalmic segment of the right internal carotid artery. **(A–C)** Large wide-necked aneurysm of the right internal carotid artery ophthalmic segment; **(D,E)** TFD implantation combined with partial coil embolization; **(F)** complete aneurysm occlusion confirmed on 5-month follow-up imaging.

**Figure 4:**
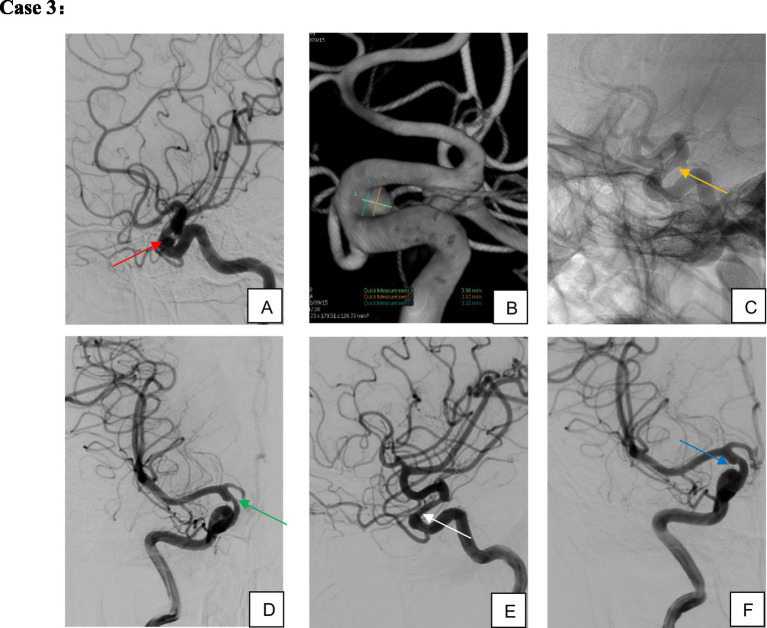
Female, 67 years old, aneurysm of the ophthalmic segment of the right internal carotid artery. **(A,B)** Right internal carotid artery ophthalmic segment wide-necked small aneurysm; **(C)** single TFD implantation; **(D,E)** four-month postoperative imaging demonstrated complete aneurysm occlusion and moderate in-stent stenosis; **(F)** without specific intervention, the in-stent stenosis spontaneously resolved on 10-month follow-up imaging while the patient remained on single-aspirin therapy.

Clinical follow-up was completed for 67 patients (including angiographic, telephone, and outpatient follow-ups), with five patients lost to follow-up (median follow-up duration: 405 days) (Figures 2–4). A favorable outcome [modified Rankin Scale (mRS) score 0–2] was achieved in 97.01% of patients, with 65 patients having mRS 0–2 and 2 patients scoring >2. Detailed outcomes are presented in Table 6.

**Table 6:** Clinical outcomes (*n* = 67).

Clinical outcomes	(*n*, %)
Cerebral infarction	2 (2.98)
TIA	2 (2.98)
Cerebral hemorrhagic complications	3 (4.48)
Bleeding at other sites	5 (7.46)
mRS score at the final follow-up	
0	46 (68.66)
1	15 (22.39)
2	4 (5.97)
3	2 (2.99)

## 6 Discussion

Intracranial complex aneurysms pose substantial challenges for endovascular therapy. Traditional techniques such as single/double stent-assisted coil embolization (SAC) or balloon-assisted coil embolization (BAC) entail intricate procedural maneuvers and are associated with relatively high long-term recurrence rates (8). In contrast, flow diverters (FDs) achieve profound hemodynamic modification by enhancing stent metal coverage and mesh density, thereby attenuating turbulent flow within the aneurysm sac. Concurrently, FDs facilitate endothelial repair and structural remodeling of the parent artery, ensuring vessel patency, preserving major collateral networks, and enabling durable aneurysm occlusion (9–11). This paradigm shift—from focal aneurysm obliteration to holistic parent artery reconstruction—has established FDs as a cornerstone in managing complex intracranial aneurysms (12–16). Landmark studies underscore their efficacy: the PUFs trial reported 6-month and 1-year complete occlusion rates of 73.6 and 86.8% in 107 large/giant aneurysms, while PREMIER demonstrated an 81.9% complete occlusion rate (113/141) and a 2.1% morbidity-mortality rate in small-to-medium aneurysms (mean size 5.0 mm), highlighting the robust safety-efficacy profile of flow diversion.

The Tubridge flow diverter (TFD), a domestically developed FD approved for clinical use in China in 2018, has shown preliminary safety and efficacy (17, 18). However, robust evidence on its performance across diverse aneurysm subtypes, optimal complication management, and long-term outcomes remains limited. A 2011 pilot study at Changhai Hospital demonstrated immediate technical success and no perioperative hemorrhagic/ischemic events but lacked extended follow-up data (19). Our study, including 75 aneurysms (median maximum diameter 4.20 mm), expands on this by showing that small-to-medium aneurysms (<10 mm) treated with standalone TFD or TFD-coiling achieved comparable final complete occlusion rates. Simple TFD implantation suffices for these lesions, streamlining procedures by eliminating complex microcatheter shaping maneuvers, reducing the risk of intraoperative aneurysm rupture during embolization, and enhancing procedural safety. Conversely, TFD-coiling is recommended for aneurysms ≥10 mm due to three key benefits: (1) coils protect complex anatomies (e.g., bleb-bearing or dissecting aneurysms), promote intrasaccular thrombosis, and mitigate postprocedural rupture risk; (2) they stabilize the FD in large wide-necked lesions, reducing stent migration or dislocation; and (3) they assist in concurrent parent artery reconstruction (20). Notably, non-saccular aneurysms treated with TFD exhibited a higher short-to-medium-term complete occlusion rate (92.86%, 13/14) compared to saccular counterparts (64.29%, 27/42), underscoring TFD’s efficacy in non-saccular pathologies.

Hemorrhagic complications associated with FDs—including delayed intraparenchymal hemorrhage (DIPH), delayed aneurysm rupture (DAR), and thrombosis-related branch vessel occlusion or in-stent stenosis–induced ischemia—remain critical considerations (21). The PED study identified large aneurysms (>10 mm) as a robust predictor of postoperative DAR, with 95% of delayed ruptures occurring in the early postoperative period (22). Zhou et al. (23) further highlighted that large/giant aneurysms, lesions on the convex side of vascular tortuosity, and those with intra-aneurysmal jet signs increase DAR risk, which coils may mitigate by dampening flow impact. In our cohort, no DAR occurred in ≥10 mm aneurysms treated with TFD-coiling, supporting this strategy for high-risk lesions. Non-aneurysm hemorrhagic events—including one parieto-occipital hemorrhage, two localized frontal subarachnoid hemorrhages, and five minor bleeding episodes (gingival/epistaxis)—were attributed to DAPT, emphasizing the need for balanced antithrombotic regimens.

Ischemic risks correlate with patient-specific factors, aneurysm characteristics, and antiplatelet strategies (24). Kallmes et al. (25) reported a 4.7% ischemic stroke rate with FDs, primarily within 30 days and more common in posterior circulation aneurysms, with the lowest incidence (2.7%) in <10 mm internal carotid artery (ICA) lesions. Notably, internal carotid artery (ICA) aneurysms <10 mm exhibited the lowest incidence (2.7%), with a demonstrated size-dependent association between ischemic stroke and aneurysm dimensions. Our study observed no acute thrombotic events, though one ICA C6 segment aneurysm developed in-stent occlusion and infarction on postoperative day 3, likely due to inadequate stent apposition—a known risk factor for in-stent stenosis or thrombosis (26), necessitating techniques like balloon remodeling or guidewire manipulation to optimize TFD deployment. Mühl-Benninghaus et al. (27) found that in-stent stenosis was observed in all cases treated with FDs, but the average degree of stenosis significantly improved from 30% in short-term follow-up to 12% in long-term follow-up. It is considered that when the stenosis degree is <25%, it is an intimal hyperplasia response within the stent, and in-stent stenosis is usually asymptomatic. However, patients with in-stent stenosis found during follow-up must undergo close clinical and angiographic monitoring. John et al. (28), reported a 10% stent stenosis rate in FD-treated aneurysms, comparable to our findings (5.45%, 3/55), where most stenoses were mild. One severe case, caused by intraoperative proximal stent migration into the aneurysm, remained stable over 2 years due to robust collateral compensation. Two moderate stenoses improved spontaneously on follow-up without intervention, reinforcing the safety of expectant management for asymptomatic lesions.

In conclusion, TFD achieved a 70.49% complete occlusion rate, 80.33% successful occlusion rate (OKM C/D), 139-day median occlusion time, and 97.01% favorable clinical outcome (mRS 0–2) in unruptured complex aneurysms. Safety and efficacy are optimized through judicious stent selection, meticulous intraoperative apposition, rigorous DAPT, and long-term medication adherence. Asymptomatic mild-to-moderate in-stent stenosis may be conservatively managed with serial imaging. These findings support TFD as a valuable option in the endovascular armamentarium for complex aneurysms, warranting further large-scale, longitudinal investigations.

### 6.1 Limitations

This study has several limitations. First, its retrospective design may be subject to selection bias and a relatively short follow-up duration. Second, the sample size was insufficient for definitive conclusions, particularly as subgroups such as the ≥10 mm aneurysm cohort included no subjects with failed successful occlusion, and the study population lacked racial diversity. These findings provide a foundational statistical basis for future large-scale, prospective investigations.

## 7 Conclusion

At the final follow-up, the Tubridge flow diverter (TFD) achieved a complete occlusion rate of 70.49% and a successful occlusion rate of 80.33% for unruptured intracranial complex aneurysms, with a median time to aneurysm occlusion of 139 days and a favorable clinical outcome rate (mRS 0–2) of 97.01%. Appropriate stent selection, adequate intraoperative stent apposition, optimal perioperative antiplatelet therapy, and long-term medication adherence effectively reduce ischemic complications. TFD is safe and effective for treating unruptured intracranial complex aneurysms, supporting its continued use in clinical practice.

## Data Availability

The original contributions presented in the study are included in the article/supplementary material, further inquiries can be directed to the corresponding author.

## Footnotes

^1^ www.zstats.net
